# Assessing the impact of moxibustion on colonic mucosal integrity and gut microbiota in a rat model of cerebral ischemic stroke: insights from the “brain-gut axis” theory

**DOI:** 10.3389/fneur.2025.1450868

**Published:** 2025-02-27

**Authors:** Yi-Xia Ding, Liang-Liang Chen, Kui-Wu Li, Ling Zou, Lu-Min Liao, Xiao-Yu Han, Jie OuYang, Yue-Ping Wu, Wen-Dong Zhang, Hao Ran Chu

**Affiliations:** ^1^Department of Encephalopathy (V), The Second Affiliated Hospital of Anhui University of Chinese Medicine (Anhui Acupuncture Hospital), Hefei, Anhui, China; ^2^Institute of Clinical Acupuncture and Moxibustion, Anhui Academy of Chinese Medicine, Hefei, Anhui, China; ^3^Anhui Clinical Medical Research Center of Acupuncture and Moxibustion, Hefei, Anhui, China; ^4^Department of Spleen and Stomach Diseases, The Second Affiliated Hospital of Anhui University of Chinese Medicine (Anhui Acupuncture Hospital), Hefei, Anhui, China; ^5^Graduate School of Anhui University of Chinese Medicine, Hefei, Anhui, China; ^6^Outpatient Department, The Second Affiliated Hospital of Anhui University of Chinese Medicine (Anhui Acupuncture Hospital), Hefei, Anhui, China

**Keywords:** brain-gut axis, cerebral ischemic stroke, gut microbiota, intestinal mucosal barrier, moxibustion

## Abstract

**Objective:**

The aim of this study is to assess the impact of moxibustion on the colonic mucosal barrier and gut microbiota in a rat model of cerebral ischemic stroke (CIS).

**Method:**

The CIS rat model was established using the modified Zea Longa suture method. Successfully modeled rats were randomly allocated into a model group and a moxibustion group, with a sham surgery group serving as the control. The moxibustion group received suspended moxibustion at Dazhui (GV 14), Baihui (GV 20), Fengfu (GV 16), and bilateral Tianshu (ST 25) and Shangjuxu (ST 37) acupoints. Neurological function was assessed using the Longa score, and brain infarct size was assessed through 2,3,5-triphenyl tetrazolium chloride staining. Gut microbiota composition was analyzed using 16S rDNA amplification sequencing. Intestinal mucosal permeability was evaluated using the FITC-Dextran tracer method. The serum ET-1 levels and the expression of Occludin and ZO-1 proteins in colonic tissues were also measured.

**Result:**

The model group exhibited significantly higher Longa scores, larger brain infarct size, and higher serum FITC-Dextran levels and ET-1 levels when compared with the sham surgery group (*p* < 0.01). The model group demonstrated decreased expression of Occludin and ZO-1 in colonic tissues (*p* < 0.01) and changes in gut microbiota structure. When compared to the model group, the moxibustion group demonstrated significantly lower Longa scores, smaller brain infarct size, and lower serum FITC-Dextran levels and ET-1 levels (*p* < 0.05). Furthermore, the moxibustion group demonstrated decreased inflammatory cell infiltration in colonic tissues, increased expression of Occludin and ZO-1 proteins in colonic tissues (*p* < 0.05), enhanced gut microbiota structure, and a decreased Simpson index (*p* < 0.05).

**Conclusion:**

Moxibustion can improve the neurological dysfunction in CIS model rats. The mechanism may be associated with the improvement in gut microbiota dysbiosis, reduction in colonic mucosal permeability, and restoration of intestinal mucosal barrier damage.

## 1 Introduction

Stroke is the second leading cause of death globally, with cerebral ischemic stroke (CIS) accounting for 62.4% of all stroke cases (1). CIS is characterized by a clinical syndrome resulting from inadequate blood supply to the vessels of the brain, leading to ischemia and hypoxia in the affected brain tissue, which causes neuronal damage and a series of brain function impairments (2).

The “brain-gut axis” has recently gained significant attention, with its role in CIS prevention and treatment gaining widespread recognition (3, 4). The gut microbiota, a crucial regulator of the brain-gut axis, influences the physiological and pathological states of both the gut and brain through neural, immune, and endocrine pathways (5). Patients post-stroke often experience gut microbiota dysbiosis, increased intestinal mucosal permeability, decreased expression of tight junction proteins, and damage to the mucosal barrier. These changes can lead to bacterial translocation and immune-inflammatory responses, further exacerbating neurological damage (3). Consequently, targeting the gut microbiota presents a promising therapeutic strategy for CIS (6–8).

Moxibustion therapy has demonstrated efficacy in enhancing neurological function and reducing complications in CIS; however, its underlying mechanisms remain elusive. Therefore, we propose the hypothesis that moxibustion intervention can improve CIS nerve function damage by regulating intestinal microbiota structure and improving intestinal barrier damage. The objective of this study is to investigate the impact of moxibustion on neurological function impairment and gut microbiota in a rat model of CIS, and to preliminarily assess its mechanistic pathways.

## 2 Materials and methods

### 2.1 Experimental animals

Forty-two SPF-grade male Sprague–Dawley (SD) rats, weighing 200–250 g, were provided by Pizhou Oriental Breeding Co., Ltd. [License No. SCXK (Suzhou) 2017–0003]. The rats were housed at the Experimental Animal Center of Anhui University of Chinese Medicine under controlled conditions, including room temperature maintained at 22°C–26°C, relative humidity ranging from 50–70%, with no restrictions on food and water and exposure to natural lighting. Following a 7-day acclimatization period, the formal experiment was initiated.

### 2.2 Experimental drugs and reagents

The following materials were used in this study: moxa sticks sourced from Yueyang Aijiantang Biotechnology Co., Ltd. (Diameter 0.7 cm, Length 12 cm, Batch No. A190929); 2,3,5-triphenyl tetrazolium chloride (TTC) obtained from Beijing Solarbio Science and Technology Co., Ltd. (Batch No. 20210525); FITC-Dextran 40 K procured from Xi’an Ruixi Biological Technology Co., Ltd. (Batch No. 20210511); endothelin-1 (ET-1) enzyme-linked immunosorbent assay (ELISA) kit supplied by Wuhan JYMBio Technology Co., Ltd. (Batch No. GR2021-08); Trizol purchased from Life Technologies (Batch No. 251804); PCR primers for ZO-1 and Occludin obtained from Sangon Biotech (Shanghai) Co., Ltd.; SYBR Green fluorescent dye PCR detection kit provided by Novoprotein (Batch No. 0516511); reverse transcription kit sourced from TaKaRa (Batch No. AJ51485A); ZO-1 antibody purchased from Abcam (Batch No. ab216880); Occludin antibody obtained from Bioss (Batch No. ad03131638); goat anti-mouse IgG antibody, goat anti-rabbit IgG antibody, and GAPDH antibody procured from Zsbio (Batch No: 140193, 202,700,514, and 200,040,908, respectively); and MP stool genomic DNA extraction kit acquired from MP Bio, United States.

### 2.3 Group assignment and model preparation

Forty-two rats were randomly allocated, with 12 rats assigned to the sham surgery group and the remaining 30 rats used to induce the CIS model via the modified Zea Longa suture method. Following modeling, 4 rats died and 2 failed to achieve successful modeling, necessitating their exclusion. The remaining 24 rats were randomly distributed into the model group and the moxibustion group, comprising 12 rats each. All procedures involving animal handling in this study strictly adhered to the Guiding Opinions on Treating Experimental Animals Humanely issued by the Ministry of Science and Technology of China in 2006 and received approval from the Ethics Committee of Anhui University of Chinese Medicine (Ethics No. AHUCM-rats-2021054).

The right-sided cerebral ischemia model in rats was induced using the modified Zea Longa suture method (9). Rats in the model group were anesthetized via intraperitoneal injection of sodium pentobarbital (2%, 45 mg/kg). A 2 cm longitudinal incision was made 2–3 cm to the right of the midline of the neck to expose and isolate the right common carotid artery (CCA), external carotid artery (ECA), and internal carotid artery (ICA). The proximal end of the CCA and ECA were ligated with sutures, and the ICA was clamped. A “V”-shaped small incision was made approximately 1 cm from the bifurcation of the CCA, ECA, and ICA using micro scissors. The suture was inserted into the ICA, and the arterial clamp on the ICA was released, allowing the suture to be inserted to a depth of 18–20 mm. The incision was closed layer by layer, and excess sutures were removed. In the sham surgery group, the blood vessels were isolated without inserting the suture. Twenty-four hours post-modeling, rats were assessed using the modified Zea Longa 5-point scoring standard upon awakening from anesthesia (10). Rats scoring 1–3 points were considered successfully modeled and included in the experiment.

### 2.4 Intervention methods

Following successful modeling, the moxibustion group received mild moxibustion intervention. The selected acupoints included Dazhui (GV 14, Between the 7th cervical vertebra and the 1st thoracic vertebra, in the center of the back), Baihui (GV 20, Middle of the parietal bone), Fengfu (GV 16, The depression on the back of the occipital atlantoaxial joint behind the occipital crest), and bilateral Tianshu (ST 25, The intersection point of lower 1/4 and upper 3/4 of the sternoclavicular symphysis and pubic symphysis is the rat navel, 5 mm away from the navel and at the navel level) and Shangjuxu (ST 37, Posterolateral knee joint, about 10 mm below the small head of fibula). The location and method of acupoint selection refer to the literature (11). The rats were fixed in a supine position on a moxibustion rack to restrict movement. ST 25 and ST 37 were selected bilaterally. Specially prepared fine moxa sticks were ignited and positioned approximately 2 cm from the acupoints, with a moxibustion acupoint hole diameter of 0.5 cm. Moxibustion sessions were administered once daily for 20 min each specific acupoint, over a span of 7 consecutive days. Both the sham surgery group and the model group rats were fixed in a supine position on the moxibustion stand to restrict movement once daily for 20 min each over seven consecutive days. Other than that, no additional intervention was accepted.

### 2.5 Sample collection

Following the experimental treatments, the rats were subjected to fasting while having access to water. Fresh fecal pellets (3–4 per rat) were collected and placed in sterile centrifuge tubes and preserved at −80°C. Four rats from each group were randomly chosen and euthanized via rapid decapitation. The entire brain tissue was promptly extracted and frozen at −20°C for 20 min for TTC staining. The remaining 8 rats were administered 0.4 mg/g FITC-Dextran via gavage 20 h prior to sample collection. Blood (4–5 mL) was obtained from the abdominal aorta, allowed to clot at room temperature for 20 min, and then centrifuged at 4°C, 3,000 r/min for 15 min to obtain serum, which was stored at −80°C. Subsequent to blood collection, a 2–3 cm segment of the colon, situated 7–8 cm from the anus, was procured, cleansed, and partitioned into three segments: one segment was stored in freezing tubes at −80°C, and the remaining two were fixed in 4% paraformaldehyde.

### 2.6 Observation indicators and detection methods

(1) Neurological function scoring: upon awakening from anesthesia, the Longa five-point scale was used to assess the neurological function of rats.

(2) TTC method for assessing rat brain infarct area: the entire brain was extracted and sliced along the coronal plane into 5 sections, each approximately 2 mm in thickness. These sections were then immersed in 2% TTC solution at 37°C for 30 min in darkness, followed by fixation for 24 h. Subsequently, photographs were captured. Areas exhibiting normal tissue appeared red, whereas infarcted areas appeared white. Infarct area analysis was performed using Image-Pro Plus 6.0 software, with the total infarct area on each section representing the infarct area on one side of the brain.

(3) Intestinal mucosal permeability detection: FITC-Dextran tracing was used to evaluate intestinal mucosal permeability in rats. Serum samples were collected, and fluorescence intensity was assessed using a microplate reader, with excitation and emission wavelengths of 488 nm and 525 nm, respectively.

(4) ELISA method for detecting ET-1 levels in serum: Serum samples were appropriately diluted and underwent sequential steps including sample injection, incubation, and plate sealing. Subsequently, color reagent A and B were added in sequence, followed by thorough mixing by shaking, and the mixture was then incubated in darkness at 37°C for 15 min. The reaction was stopped by adding a stop buffer. The absorbance (OD value) at 450 nm was measured using a microplate reader.

(5) Histopathological changes in rat colon mucosa tissues observed by hematoxylin and eosin staining: Colonic tissues were collected and subjected to standard deparaffinization using gradient ethanol, followed by rinsing with running water. Differentiation was carried out using 1% hydrochloric acid alcohol, followed by additional rinsing with running water. Following sealing of the slices with neutral gum, the morphology of colonic tissues was examined under a microscope.

(6) Western blot for detecting Occludin and ZO-1 protein expression in rat colonic tissues: Colonic tissues (100 mg) were homogenized and lysed. Following centrifugation, the supernatant containing proteins was collected. Protein concentration was determined using a BCA kit. Subsequently, proteins were separated by polyacrylamide gel electrophoresis and transferred to a PVDF membrane. The membrane was blocked at room temperature for 1 h. Primary antibodies against Occludin, ZO-1, and β-actin were added and incubated overnight. The following day, appropriate secondary antibodies were added and incubated at room temperature for 1 h. ECL reagent was used for exposure and development. Band intensity was quantified using ImageJ software, with GAPDH serving as the internal control protein.

(7) Real-time fluorescence quantitative PCR for detecting Occludin and ZO-1 expression in colonic tissues of rats: Colonic tissues (100 mg) were weighed, and total RNA was extracted. The reaction system comprised of 2.5 μL of 2× mixture, 2 μL of gene primers, 1 μL of cDNA, and 2 μL of ddH_2_O. The amplification conditions were as follows: pre-denaturation at 95°C for 30 s, followed by denaturation at 95°C for 10 s, and annealing/extension at 60°C for 30 s, for a total of 40 cycles. A melting curve was generated by increasing the temperature from 60°C to 95°C at a rate of 0.3°C every 15 s. Relative expression of the target genes was calculated using the 2^−△△CT^ method with β-actin as the internal reference. The primer sequences are provided in Supplementary Table S1.

(8) Immunohistochemical detection of ZO-1 and Occludin protein expression in colonic tissues: paraffin-embedded colonic tissue sections underwent baking, xylene immersion, gradient ethanol deparaffinization, and high-pressure antigen retrieval. After treatment with 3% H_2_O_2_ and PBS washing, primary antibodies (ZO-1, 1:200; Occludin, 1:200) were applied, and sections were incubated at 37°C for 60 min, followed by three PBS washes. Subsequently, secondary antibodies (goat anti-mouse IgG, 1:400; goat anti-rabbit IgG, 1:400) were applied and the sections incubated at 37°C for 20 min. After PBS washing, DAB was used for color development, which was then discontinued upon observation of positive staining under a microscope. The positive expression of ZO-1 and Occludin proteins in colonic tissues was quantified using the JD801 morphometric image analysis system, and average absorbance values were calculated.

(9) Intestinal microbial community sequencing by 16S rDNA amplification sequencing: Total bacterial DNA was extracted from rat feces, and the V3-V4 region was subjected to PCR amplification. After addition of sample-specific tags, libraries were constructed and assessed using the Agilent 2,100 Bioanalyzer. The sequencing library was accurately quantified, and high-throughput sequencing was conducted using the Illumina NovaSeq 6,000 platform using a 2 × 250 bp paired-end sequencing strategy. Data were processed with QIIME2 software for quality filtering, denoising, merging, and chimera removal, resulting in a Feature table and representative sequences. Clustering was performed to generate operational taxonomic units (OTUs). Each OTU representative sequence was aligned against bacterial databases using QIIME2 for taxonomic annotation. Analyses of OTU abundance, α-diversity, β-diversity, and LEfSe differential analysis were carried out using QIIME2.

### 2.7 Statistical analysis

Statistical analysis was conducted using SPSS 26.0 software. Measurement data are presented as mean ± standard deviation (x̅ ± s). One-way analysis of variance (ANOVA) was used for datasets exhibiting normal distribution and homogeneity of variance, followed by *post hoc* LSD test for pairwise comparisons between groups. In cases where variances were not homogeneous, Tamhane’s T2 method was used. A significance level of *p* < 0.05 was deemed statistically significant.

## 3 Results

### 3.1 Comparison of relative infarct area and Longa score in rats in each group

Rats in the sham surgery group exhibited no neurological impairment, and TTC staining indicated the absence of infarct lesions. When compared to the sham surgery group, the model group demonstrated increased neurological damage scores and relative infarct volume (*p* < 0.01). Conversely, when compared to the model group, the moxibustion group displayed reduced neurological damage scores and relative infarct volume (*p* < 0.05). For further details, refer to Table 1 and Supplementary Figure S1.

**Table 1:** Comparison of the relative infarct size and Longa score of rats in each group (***x̄*** ± *s*).

	*n*	Infarct size (mm^2^)	*n*	Longa score (point)
Sham surgery group	4	0	12	0
Model group	4	15.434 ± 0.667^1^	12	2.50 ± 0.926^1^
Moxibustion group	4	11.094 ± 0.413^2^	12	0.75 ± 0.707^2^
*F*-value		926.434		32.237
*p-*value		<0.001		<0.001

^1p < 0.01 vs. Sham surgery group; 2p < 0.05 vs. Model group.^

### 3.2 Comparison of serum FITC-dextran and ET-1 levels in rats in each group

When compared to the sham surgery group, the model group exhibited increased levels of serum FITC-Dextran and ET-1 (*p* < 0.01). Conversely, compared to the model group, the moxibustion group demonstrated decreased levels of serum FITC-Dextran and ET-1 (*p* < 0.01). Refer to Table 2 for detailed information.

**Table 2:** Comparison of serum FITC-Dextran and ET-1 levels of rats in each group (***x̄*** ± *s*, *n =* 8 in each group).

	FITC-Dextran (OD)	ET-1 (pg/mL)
Sham surgery group	0.298 ± 0.025	50.053 ± 3.654
Model group	0.668 ± 0.026^1^	179.034 ± 40.026^1^
Moxibustion group	0.548 ± 0.016^2^	86.987 ± 11.723^2^
*F*-value	550.287	60.411
*P-*value	<0.001	<0.001

^1p < 0.01 vs. Sham surgery group; 2p < 0.01 vs. Model group.^

### 3.3 Comparison of colonic mucosal morphology in rats in each group

**Figure 1:**
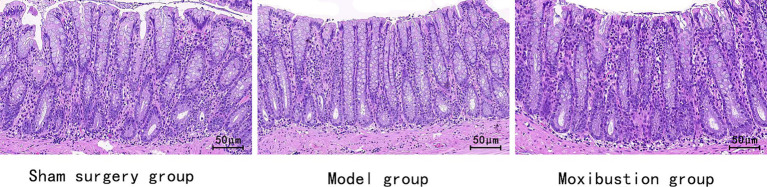
Comparative analysis of intestinal mucosa morphology in each group (HE staining, ×400).

Rats in the sham surgery group displayed normal colonic tissue structure characterized by orderly and compact arrangement of colonic epithelial cells and glands. Conversely, rats in the model group exhibited mild infiltration of inflammatory cells in colonic tissues, without any mucosal erosion, ulceration, congestion, or edema. Within the moxibustion group, a slight infiltration of inflammatory cells was observed, with no additional abnormal changes noted. Please refer to Figure 1 for visual representation.

### 3.4 Comparison of Occludin and ZO-1 protein expression levels in colonic tissues of rats in each group

When compared to the sham surgery group, the model group exhibited reduced expression levels of Occludin and ZO-1 proteins in colonic tissues (*p* < 0.01). Conversely, compared to the model group, the moxibustion group demonstrated elevated expression levels of Occludin and ZO-1 proteins in colonic tissues (*p* < 0.05). Detailed information can be found in Table 3 and Supplementary Figure S2.

**Table 3:** Comparison of Occludin and ZO-1 protein expression in rat colonic tissues (***x̄*** ± *s*).

	Occludin	ZO-1
Sham surgery group	0.560 ± 0.078	0.222 ± 0.047
Model group	0.191 ± 0.036^1^	0.088 ± 0.013^1^
Moxibustion group	0.306 ± 0.023^2^	0.149 ± 0.019^2^
*F*-value	40.247	14.618
*P-*value	<0.005	<0.001

^1p < 0.01 vs. Sham surgery group; 2p < 0.05 vs. Model group.^

### 3.5 Occludin and ZO-1 mRNA expression in colonic tissues of rats

When compared to the sham surgery group, the model group displayed reduced mRNA expression levels of Occludin and ZO-1 in colonic tissues (*p* < 0.01). Conversely, when compared to the model group, the moxibustion group exhibited increased mRNA expression levels of Occludin and ZO-1 in colonic tissues (*p* < 0.01). Please refer to Table 4 for detailed data.

**Table 4:** Comparison of Occludin and ZO-1 mRNA expression in rat colonic tissues (***x̄*** ± *s*).

	Occludin	ZO-1
Sham surgery group	1.026 ± 0.141	0.968 ± 0.078
Model group	0.345 ± 0.066^1^	0.421 ± 0.044^1^
Moxibustion group	0.597 ± 0.054^2^	0.585 ± 0.061^2^
*F*-value	104.973	160.936
*P-*value	<0.001	<0.001

^1p < 0.01 vs. Sham surgery group; 2p < 0.01 vs. Model group.^

### 3.6 Positive expression of Occludin and ZO-1 proteins in colonic tissues of rats

When compared to the sham surgery group, the model group exhibited reduced positive expression of Occludin and ZO-1 proteins in colonic tissues (*p* < 0.01). Conversely, compared to the model group, the moxibustion group demonstrated increased positive expression of Occludin and ZO-1 proteins in colonic tissues (*p* < 0.01). Detailed information is provided in Table 5 and Supplementary Figure S3.

**Table 5:** Comparison of positive protein expression of Occludin and ZO-1 in rat colonic tissues (***x̄*** ± *s*).

	Occludin	ZO-1
Sham surgery group	0.585 ± 0.023	0.605 ± 0.019
Model group	0.195 ± 0.007^1^	0.179 ± 0.011^1^
Moxibustion group	0.367 ± 0.032^2^	0.364 ± 0.044^2^
*F*-value	147.776	110.848
*P-*value	<0.001	<0.001

^1p < 0.01 vs. Sham surgery group; 2p < 0.01 vs. Model group.^

### 3.7 Analysis of gut microbiota results

#### 3.7.1 OTU Venn diagram analysis

**Figure 2:**
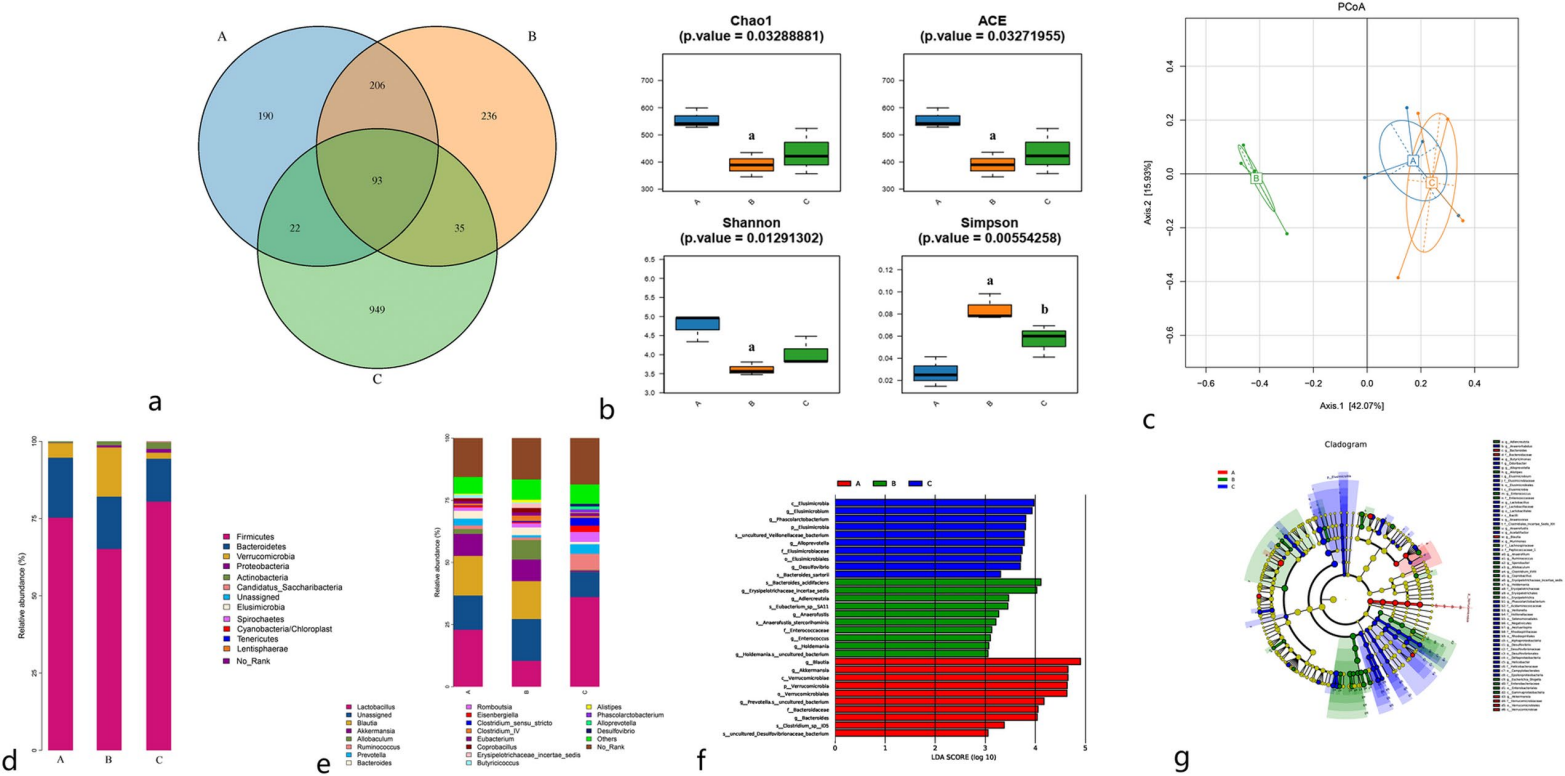
**(A)** Venn diagram of gut microbiota OTUs of rats in each group. **(B)** Comparison of *α* diversity index. **(C)** PCoA plot of *β* diversity analysis. **(D)** Species distribution of gut microbiota at the phylum level in rats in each group. **(E)** Species distribution of gut microbiota at the genus level in rats in each group. **(F)** LDA scores of the gut microbiota of rats, with significant differences between groups. **(G)** Evolutionary relationship between significantly different gut microbiota in rat feces. A: Sham surgery group; B: model group; C: moxibustion group.

As illustrated in Figure 2A, the total number of OTUs observed in the sham surgery group, model group, and moxibustion group were 511, 570, and 1,099, respectively. The unique OTUs identified in the sham surgery group, model group, and moxibustion group were 190, 236, and 949, respectively. Furthermore, comparative analysis revealed that following moxibustion intervention, the abundance of certain gut microbiota in rats increased when compared to the model group (*p* < 0.05).

#### 3.7.2 Diversity analysis

The alpha diversity analysis of gut microbiota encompasses two categories: richness indices (Chao1, Ace) and diversity indices (Shannon, Simpson). When compared to the sham surgery group, the model group exhibited reduced Chao1, Ace, and Shannon indices (*p* < 0.01) along with an increased Simpson index (*p* < 0.01). Conversely, compared to the model group, the moxibustion group displayed increased Chao1, Ace, and Shannon indices, although without statistical significance (*p* > 0.05), and decreased Simpson index (*p* < 0.05). Refer to Figure 2B.

Beta diversity analysis is used to evaluate diversity differences among various samples by analyzing the composition of diverse microbial communities, thereby contrasting the structures of different microbial communities. The principal component PCoA1 contributed 42.07%, and the secondary component PCoA2 contributed 15.93%. Notably, a significant separation was observed between the model group and both the sham surgery group and moxibustion group in the primary component (42.07%), indicating a substantial distinction in gut microbiota structure between the model group and the other two groups. The gut microbiota structure of the sham surgery group and moxibustion group exhibited closer resemblance. Refer to Figure 2C.

#### 3.7.3 Classification and composition analysis of gut microbiota

Upon comparing the relative abundance of microbial communities at the phylum level in rats from each group, Firmicutes and Bacteroidetes emerged as the dominant phyla, followed by Verrucomicrobia and Proteobacteria. When compared to the sham surgery group, the model group displayed downregulation of Firmicutes and Bacteroidetes relative abundances (*p* < 0.05), along with an upregulation of Verrucomicrobia and Proteobacteria relative abundances (*p* < 0.05). Conversely, compared to the model group, the moxibustion group exhibited upregulation of Firmicutes and Proteobacteria relative abundances (*p* < 0.05), coupled with downregulation of Verrucomicrobia relative abundance (*p* < 0.01). Refer to Figure 2D for details.

At the genus level, when compared to the sham surgery group, the model group displayed upregulation of *Clostridium* cluster IV, *Allobaculum*, and *Alistipes*, although without statistical significance (*p* > 0.05), while *Lactobacillus*, *Prevotella*, *Butyricicoccus*, and *Blautia* revealed downregulation, also without statistical significance (*p* > 0.05). Conversely, compared to the model group, the moxibustion group demonstrated upregulation of *Lactobacillus*, *Ruminococcus*, *Phascolarctobacterium*, and *Desulfovibrio* (*p* < 0.05), along with the downregulation of *Bacteroides* and *Alistipes* (*p* < 0.05). Refer to Figure 2E.

#### 3.7.4 Differential species analysis

LEfse analysis using linear discriminant analysis (LDA) was used for multi-group comparison to discern microbial taxa significantly associated with different samples. As depicted in Figures 2F,G, notable differences in microbial taxa were evident among the sham surgery group, model group, and moxibustion group, with the most significant differences observed between the model group and the sham surgery group.

## 4 Discussion

Cerebral ischemic stroke is categorized within the field of “stroke” in traditional Chinese medicine, where its primary pathological mechanisms entail disruptions in qi and blood circulation, along with cerebral blood vessel obstruction. This study is based on the brain-gut axis framework theory for moxibustion intervention. Specifically, the chosen acupoints, namely Baihui (GV 20), Fengfu (GV 16), and Dazhui (GV 14), are aligned with the principles of “dredging the governing vessels and harmonizing the mind.” The combined stimulation of these acupoints aims to facilitate vessel clearance, harmonizing the mind, restoring consciousness, and opening orifices. Also, the inclusion of the lower sea point in the large intestine, Shangjuxu (ST 37), and the alarm point Tianshu (ST 25), serve to regulate intestinal qi movement. This modulation guides obstructive pathogenic factors from the upper body to descend through the gastrointestinal tract, adhering to the therapeutic tenet of addressing upper ailments through lower interventions. Clinical reports have underscored the distinctive therapeutic efficacy of acupuncture and moxibustion therapy rooted in the brain-gut axis theory in treating CIS (12–14). The results of this study underscore that moxibustion targeting the aforementioned acupoints can significantly enhance the neurological function scores in CIS model rats and reduce the cerebral infarction areas.

Recent studies have identified gut microbiota imbalance as a potential factor in central nervous system diseases (15). The brain-gut axis is a bidirectional regulatory system involving the central nervous system, enteric nervous system, autonomic nervous system, and hypothalamic–pituitary–adrenal axis. Following CIS, pathological reactions such as immune and inflammatory responses to infarction lesions, organism stress responses, abnormal intestinal motility, and activation of the sympathetic nervous system contribute to gut microbiota dysbiosis (16). This dysbiosis activates the intestinal immune system, aggravating neuronal inflammatory damage through signal transmission along the brain-gut axis (3). Studies indicate that patients with CIS exhibit significant gut microbiota dysbiosis, with the severity of this imbalance correlating positively with the severity of CIS (17, 18).

The results of this study demonstrated a significant reduction in the Chao1, Ace, and Shannon indices, and an increase in the Simpson index in the CIS model rats, indicating a decrease in gut microbiota diversity. The PCoA plot revealed a clear separation between the model group and both the sham surgery group and the moxibustion group in the principal component, indicating substantial changes in the microbial community structure. Following moxibustion intervention, the Simpson index in the moxibustion group decreased, indicating an increase in gut microbiota diversity. Also, the PCoA plot revealed that the gut microbiota structure of the sham surgery group and the moxibustion group were similar. This similarity was characterized by an increase in the relative abundance of beneficial bacteria like *Firmicutes*, *Lactobacillus*, *Ruminococcus*, and *Phascolarctobacterium*. These findings suggest that moxibustion intervention can effectively enhance gut microbiota dysbiosis in CIS model rats, regulate the microbial community structure, and enhance microbial richness and diversity, aligning with existing research results (19).

Gut microbiota plays a crucial role in regulating intestinal mucosal barrier function (20). Gut microbiota and their metabolites can modulate mucosal barrier function by influencing proteins such as mucins in the mucus layer (21). Furthermore, many probiotics can enhance the expression of tight junction proteins such as Claudin, Occludin, and ZO-1 in intestinal epithelial cells, thereby reducing intestinal mucosal barrier permeability and strengthening mucosal barrier function (22). Tight junctions are comprised of transmembrane structural proteins such as Occludin, Claudin, junctional adhesion molecules (JAM), intracellular ZO-1 proteins, and the cytoskeleton. Following CIS, gut dysbiosis leads to the disruption of intestinal mucosal integrity, increased intestinal mucosal permeability, impaired mucosal barrier function, and bacterial translocation, resulting in systemic, low-grade inflammation (23, 24).

ET-1 is a key factor in vascular endothelial injury. Increased expression of ET-1 leads to heightened vascular permeability and exacerbates neuronal damage in cerebral infarction lesions. FITC-Dextran, a low molecular weight polysaccharide, is not normally present in the rat body, and when administered by gavage, it is not absorbed by the intestine. Therefore, the serum FITC-Dextran level is positively correlated with intestinal mucosal barrier permeability. The results of this study demonstrated significant infiltration of inflammatory cells in the colonic mucosa of CIS model rats. The expression levels of tight junction proteins Occludin and ZO-1 in the colonic mucosa were decreased, while serum levels of FITC-Dextran and ET-1 were increased. These findings indicate that increased intestinal mucosal permeability and bacterial translocation in CIS model rats were significant. Following moxibustion intervention, there was an enhancement in these indicators, indicating that moxibustion can enhance the expression levels of tight junction proteins Occludin and ZO-1 in the colonic mucosa of CIS model rats, reduce mucosal permeability, and improve intestinal mucosal barrier damage and gut dysbiosis, which aligns with relevant research results (25).

## 5 Conclusion

In summary, moxibustion intervention significantly enhanced neurological function scores, reduced the cerebral infarction area in CIS model rats, ameliorated intestinal mucosal barrier damage, regulated gut microbiota structure, and increased the richness and diversity of beneficial bacterial populations. Thus, moxibustion intervention can alleviate neurological function damage in CIS model rats, possibly through its effects on improving gut microbiota and intestinal mucosal barrier damage, exerting a neuroprotective effect via the bidirectional communication mechanism of the brain-gut axis. Given the complexity of moxibustion’s mechanisms, future research should further elucidate its specific mechanisms in intervening with CIS via brain-gut interactions, explore its effects on other related conditions such as sensory disturbance and depression by utilizing amplicon sequence variants (ASVs) and further advanced bioinformatics techniques.

## Data Availability

The raw data supporting the conclusions of this article will be made available by the authors, without undue reservation.
