# Supplementary material for: Assessing the impact of moxibustion on colonic mucosal integrity and gut microbiota in a rat model of cerebral ischemic stroke: insights from the “brain-gut axis” theory
Source: Front Neurol. 2025 Feb 27;16:1450868. doi: 10.3389/fneur.2025.1450868 (PMC11903257; doi:10.3389/fneur.2025.1450868)
Supplement: Supplementary file 1 [file Data_Sheet_1.PDF]

Supplementary Table S1: Primer sequence

Gene	Forward primer （5'→3'）	Reverse primer （5'→3'）	Amplicon Size （bp）
β-actin	CCCATCTATGAGGGTTACGC	TTTAATGTCACGCACGATTTC	150
ZO-1	CAAGGAGGTAGAGCGAGGCA	CTGGGAACTTTGTTTGAACTGG	197
Occludin	TGGGAGCCTTGACATCTTGT	GGTGCATAATGATTGGGTTTG	126

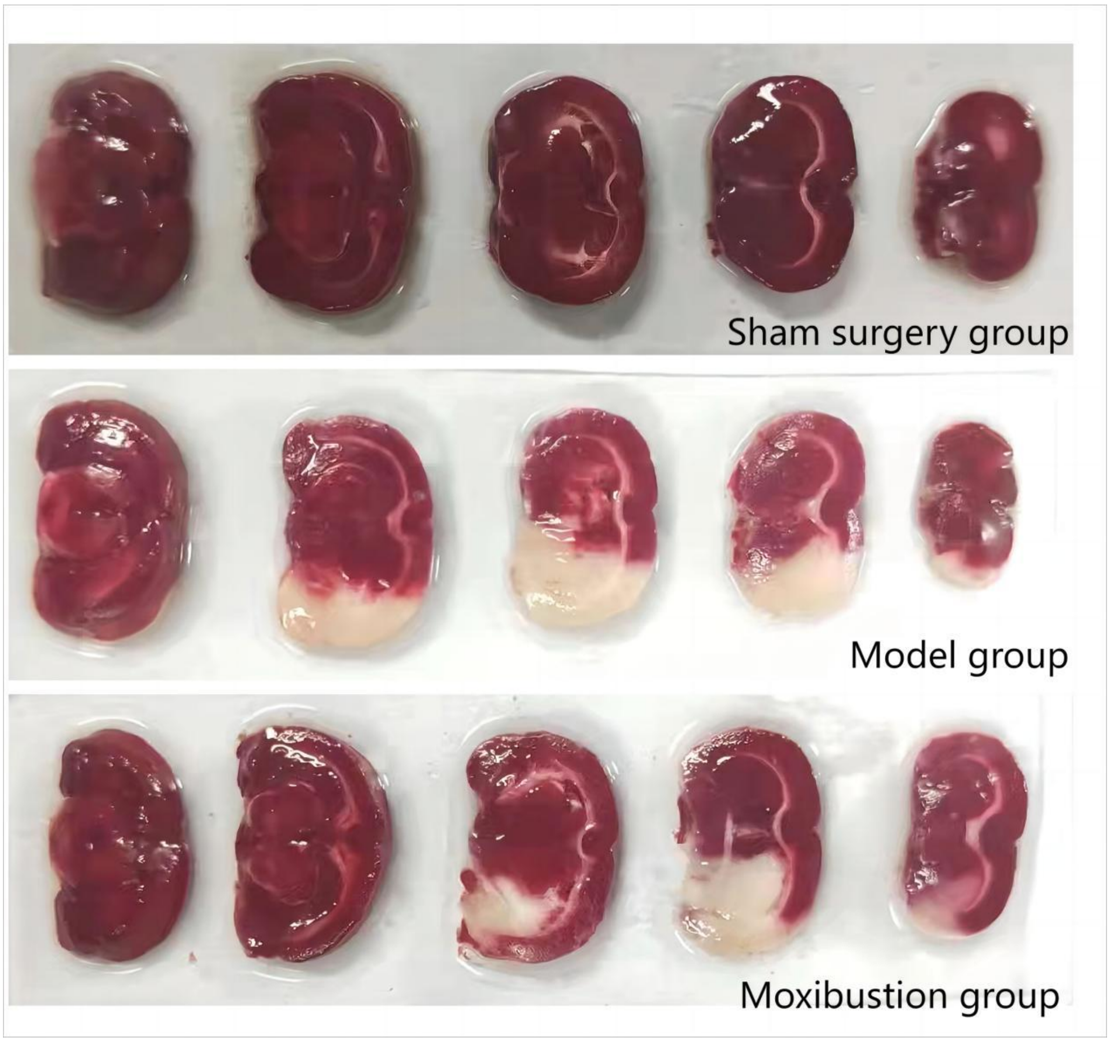

Supplementary Figure S1: Relative infarct size in each group (TTC staining, *n* = 4 in each group)

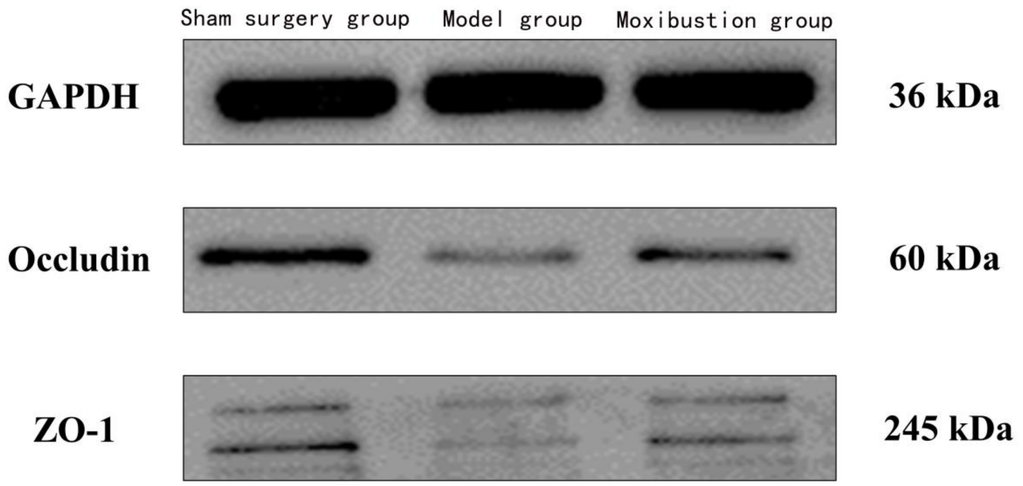

Supplementary Figure S2: Comparison of Occludin and ZO-1 protein expression in colonic tissues of rats in each group

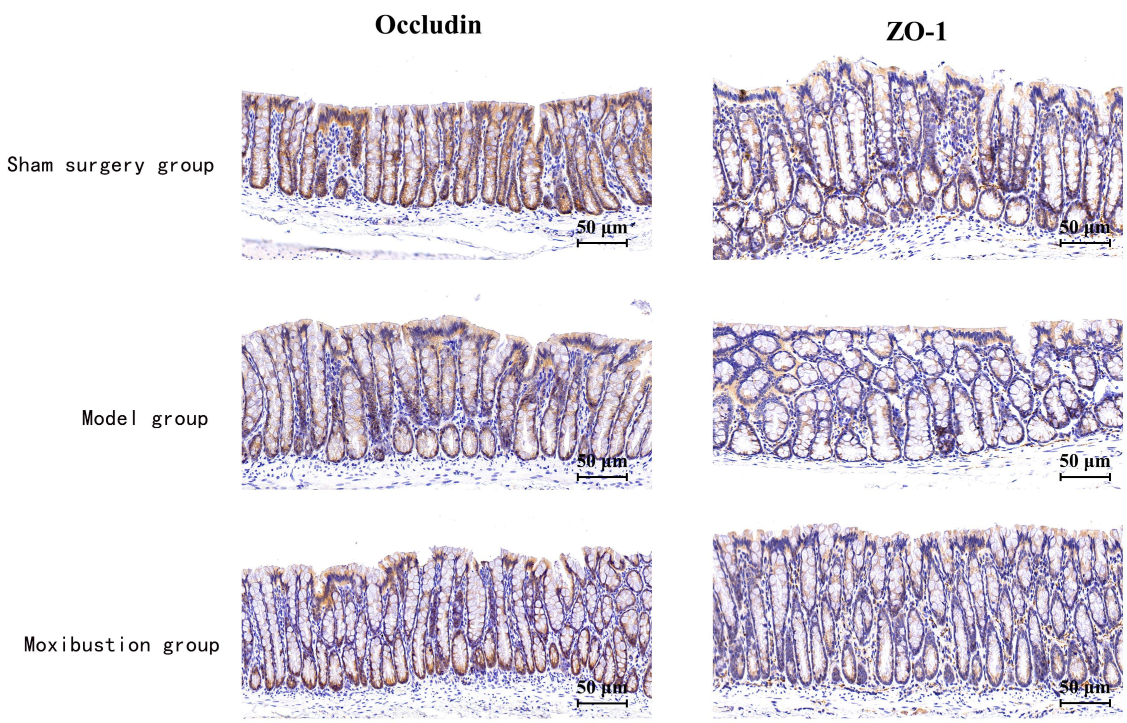

Supplementary Figure S3: Positive expression of the Occludin and ZO-1 proteins in rat colonic tissues (immunohistochemical staining, n = 8 in each group, ×400)
