# Supplementary figures and images for: Assessing the impact of moxibustion on colonic mucosal integrity and gut microbiota in a rat model of cerebral ischemic stroke: insights from the “brain-gut axis” theory
Source: Front Neurol. 2025 Feb 27;16:1450868. doi: 10.3389/fneur.2025.1450868 (PMC11903257; doi:10.3389/fneur.2025.1450868)

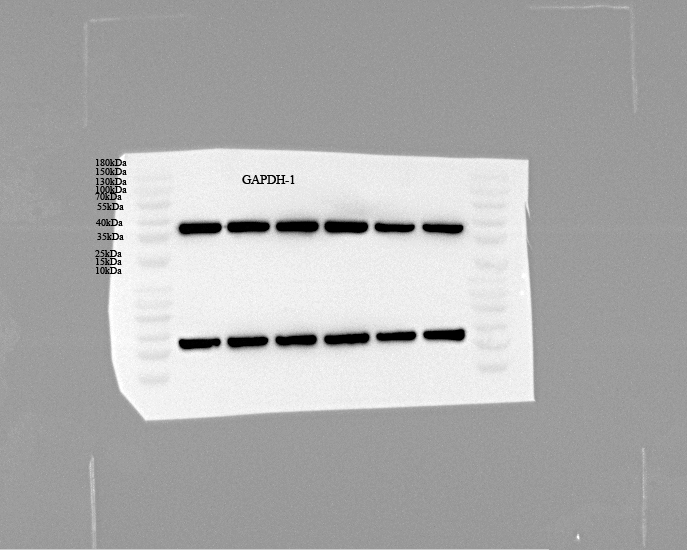

Supplement: Supplementary file 2 [file Data_Sheet_2.ZIP › high-quality images of Western Blot/丁义侠/GAPDH-1.tif]

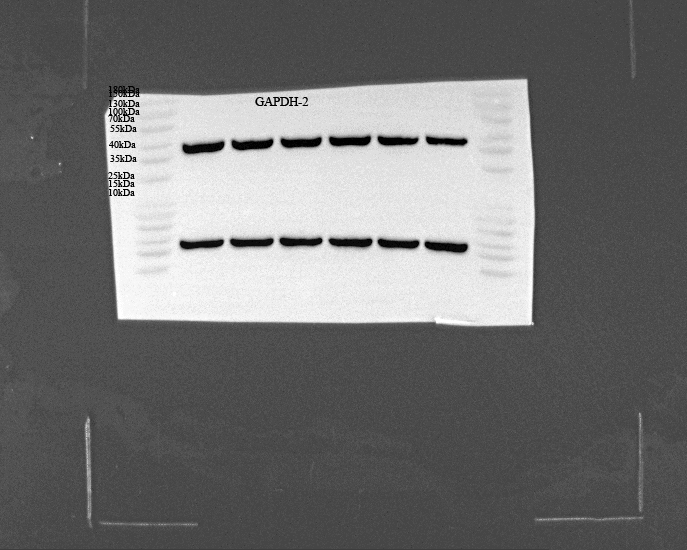

Supplement: Supplementary file 2 [file Data_Sheet_2.ZIP › high-quality images of Western Blot/丁义侠/GAPDH-2.tif]

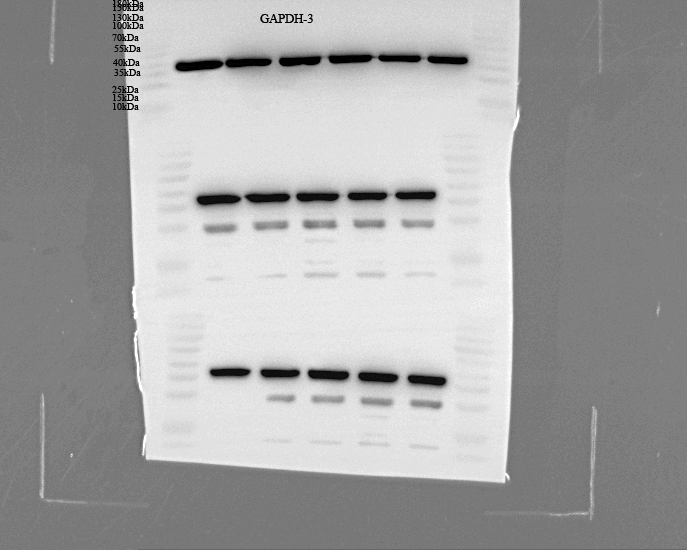

Supplement: Supplementary file 2 [file Data_Sheet_2.ZIP › high-quality images of Western Blot/丁义侠/GAPDH-3.tif]

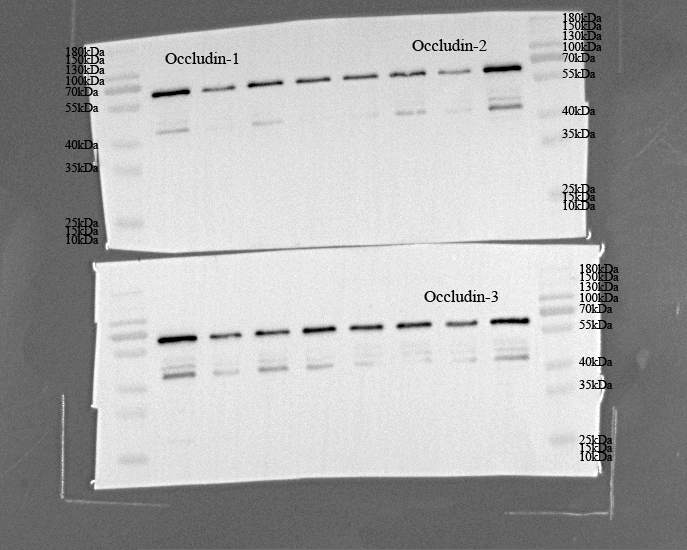

Supplement: Supplementary file 2 [file Data_Sheet_2.ZIP › high-quality images of Western Blot/丁义侠/Occludin.tif]

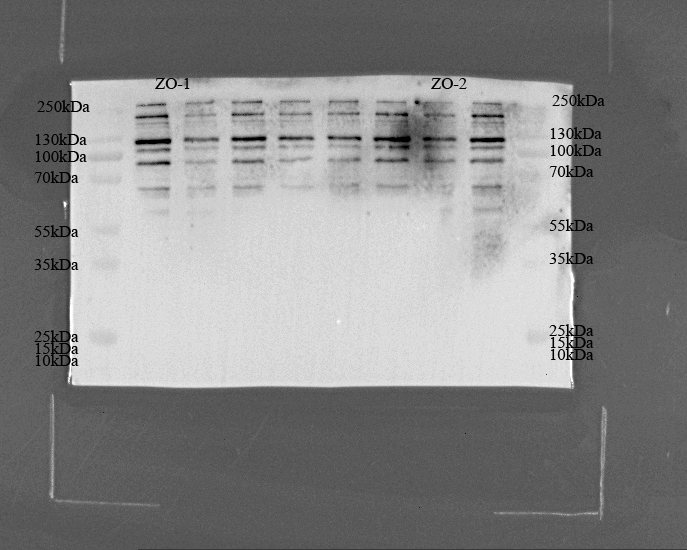

Supplement: Supplementary file 2 [file Data_Sheet_2.ZIP › high-quality images of Western Blot/丁义侠/ZO-1-2.tif]

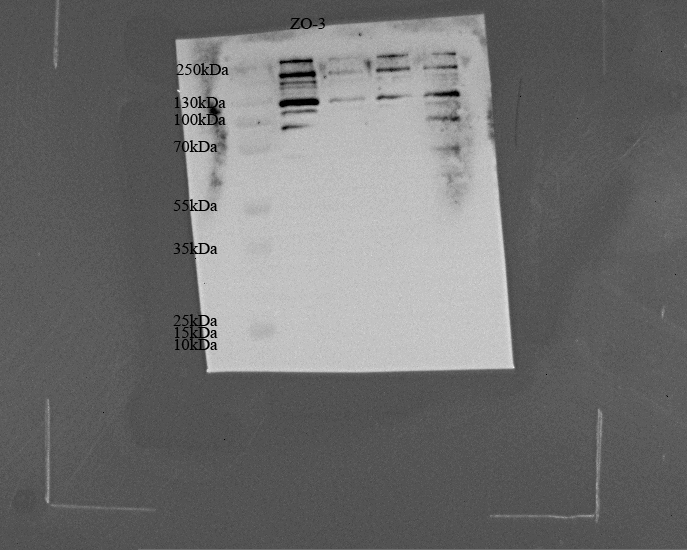

Supplement: Supplementary file 2 [file Data_Sheet_2.ZIP › high-quality images of Western Blot/丁义侠/ZO-3.tif]
